# Development of Long-Acting Human Adrenomedullin Fc-Fusion Proteins

**DOI:** 10.3390/biology11071074

**Published:** 2022-07-19

**Authors:** Sayaka Nagata, Motoo Yamasaki, Nobuko Kuroishi, Kazuo Kitamura

**Affiliations:** Frontier Science Research Center, University of Miyazaki, 5200 Kihara, Kiyotake, Miyazaki 889-1692, Japan; motoo_yamasaki@med.miyazaki-u.ac.jp (M.Y.); nobuko_kuroishi@med.miyazaki-u.ac.jp (N.K.)

**Keywords:** adrenomedullin, immunoglobulin G, hypotensive effect, recombinant protein

## Abstract

**Simple Summary:**

Adrenomedullin (AM) is a hypotensive peptide hormone that exerts anti-inflammatory effects and is involved in wound healing and embryogenesis. However, treatment requires continuous administration as the half-life of native AM is short in blood. To resolve this, we developed four human IgG1 and IgG4 Fc-fusion proteins containing full-length hAM or hAM residues 6-52 using mammalian cells. The Fc-AM produced were amidated and in the active form. All Fc-AMs stimulated cAMP production in HEK-293 cells stably expressing the AM_1_ receptor. The activities of IgG1-AM (6-52) and IgG4-AM (6-52) were higher than those of IgG1-AM and IgG4-AM. Sufficient concentrations of IgG1-AM (6-52) and IgG4-AM (6-52) were observed in blood 14 days after a single subcutaneous administration. Furthermore, after IgG1-AM (6-52) or IgG4-AM (6-52) administration, tissue transfer to the kidney and small intestine was observed. Treatment with IgG4-AM (6-52) inhibited blood pressure increase in spontaneously hypertensive rats. Fc-AM produced from mammalian cells can be easily prepared and might be an effective novel therapeutic agent.

**Abstract:**

(1) Background: Human adrenomedullin (hAM) is a hypotensive peptide hormone that exerts powerful anti-inflammatory effects. AM also had therapeutic effects in various animal experimental models of disease. However, treatment required continuous administration as the half-life of native AM is short in blood. To resolve this, we developed four human IgG1 and IgG4 Fc-fusion proteins containing full-length hAM or hAM residues 6-52. (2) Methods: We used mammalian cells to produce recombinant Fc-AM derivatives and tested the pharmacokinetics and biological activity of Fc-AM. (3) Results: We developed four Fc-fusion AMs (Fc-AM), which are long-acting AM derivatives in mammalian cells. Fc-AM had a prolonged half-life in blood and retained its ability to bind to the AM_1_ receptor. Fc-AM (6-52) induced higher cAMP levels for the receptor than Fc-AM. After the administration of IgG1-AM (6-52) or IgG4-AM (6-52) to rats, tissue transfer to the kidney and small intestine was observed. In addition, treatment with IgG4-AM (6-52) inhibited blood pressure increase in spontaneously hypertensive rats. (4) Conclusions: Fc-AM produced from mammalian cells can be easily prepared and might be an effective novel therapeutic agent.

## 1. Introduction

Human adrenomedullin (hAM) was originally isolated from a human pheochromocytoma by monitoring cyclic adenosine monophosphate (cAMP) in rat platelets [1]. AM consists of 52 amino acid residues with an amidated C-terminus and a ring structure formed by intramolecular disulfide bonds. Both of these structures are necessary for binding to receptors and to exert their biological functions. AM has pluripotent bioactivities, including vasodilatation, hormone secretion, wound healing, and anti-inflammation, which were associated with its beneficial pharmacological effects observed in various animal disease models, including inflammatory bowel disease [2], ischemic heart disease [3], sepsis [4], and stroke [5]. However, native AM has poor pharmacokinetic properties due to its rapid clearance and proteolytic degradation. Therefore, long-acting AM derivatives are desirable for the clinical application of AM.

In previous studies, we showed that AM was rapidly digested by thrombin in serum [6]. The main product is AM (13-44), which suggests thrombin is a trypsin-like protease that recognizes the Arg residues, Arg-12 and Arg-44, in AM. Therefore, we prepared AM analog in which Arg-44 was replaced by Ala ([44Ala] AM (13-52)) [6]. [44Ala] AM (13-52) was resistant to thrombin and showed comparable biological activity to native AM. Although the bioavailability of [44Ala] AM (13-52) was significantly improved, the half-life of [44Ala] AM (13-52) was comparable to that of AM, indicating it was inadequate as a therapeutic agent for chronic diseases.

To improve the pharmacokinetic properties, we developed polyethylene glycol (PEG) -conjugated AM (PEG-AM) [7] and human serum albumin-conjugated AM [8]. The amino-terminal region of AM does not contribute to the biological activity of AM and we previously conjugated large molecules, such as PEG and human serum albumin, to the N-terminal of AM. These molecules had much longer half-lives and bioavailability. However, both AM derivatives are costly as they require the chemical synthesis of the AM. Furthermore, PEG is not an endogenous molecule in humans, and although some pegylated drugs have been investigated clinically, there were reports of antibody production against them.

A non-neutralizing anti-AM antibody, adrecizumab, binds to the N-terminal region of endogenous AM in blood and causes a long-lasting increase in its levels [9]. Therefore, adrecizumab is expected to enhance the beneficial effects of endogenous AM in patients with high plasma levels of AM. Adrecizumab reduced vascular leakage and organ dysfunction, and improved survival in several models of sepsis [10]. However, phase 2a clinical trials of adrecizumab in patients with septic shock failed to demonstrate any benefit [11].

On the basis of these studies, we previously produced Fc-fusion AM by genetic recombination using *Escherichia coli* [12]. However, recombinant proteins formed inclusion bodies (insoluble fraction) that required cell crushing and refolding [13]. Furthermore, the C-termini of recombinant proteins produced by *E. coli* are not amidated. Therefore, AM produced in *E. coli* requires refolding and amidation, as well as purification at each step. IgG Fc-fusion proteins produced in mammalian cells have the advantage that refolding or amidation are not required [14]. In the present study, we developed a long-acting IgG Fc-fusion hAM using mammalian cells.

## 2. Materials and Methods

### 2.1. Cloning and Production of Recombinant Proteins

Genes encoding recombinant proteins were designed using GENETYX Ver.13 (GENETYX CORPORATION, Tokyo, Japan). Genes encoding the IgG1 or IgG4 Fc region and AM or AM (6-52) were connected with a sequence encoding a (GGGGS)3 linker. The amino terminus of AM or AM (6-52) were attached to the Fc region as the carboxy end of AM is essential for biological activity. The structures of AM-IgG4 and AM-IgG1 were previously described in the Supplementary Data [12]. The cloning was performed using an In-Fusion HD Cloning Kit (Takara, Shiga, Japan).

Stellar Competent cells (Takara, Shiga, Japan) were transformed with the ligation mixtures and single bacterial colonies were obtained after overnight growth on LB- based agar medium with Zeocin (Fast-Media Zeo Agar: InvivoGen, San Diego, CA, USA).

The recombinant plasmids, pFUSEN-IgG1 Fc-AM, pFUSEN-IgG1 Fc-AM (6-52), pFUSE-IgG4 Fc-AM, and pFUSE-IgG4 Fc-AM (6-52) were confirmed by PCR and DNA sequencing. The plasmids used in this study are transient expression vectors. The 1L of Expi293F cells based on human embryonic kidney (HEK293) cells (2.5 × 10^6^ cells/mL) were transformed with 1.0 mg of these plasmids and expression was induced using an enhancer kit (NeoFectionEN-1) (Astec Cell Science Research Laboratory, Fukuoka, Japan). After free floating culturing for 5 days, supernatants were collected and the recombinant proteins were purified from the medium by protein A affinity chromatography (HiTrap Protein A HP: GE Healthcare, Uppsala, Sweden). The purification was performed according to the manufacturer’s manual: 30 mL of supernatant was filtered through a 0.22 μm filter. The obtained eluate was applied to 1 mL of HiTrap Protein A HP and washed with 10 mL of Binding Buffer (20 mM sodium phosphate, pH 7.0) and eluted with elution buffer (0.1 M glycine-HCl, pH 2.7). The eluate was immediately neutralized with neutralizing buffer (1.0 M Tris-HCl, pH 9.0).

The molecular weights of recombinant proteins were estimated by SDS-PAGE. In addition, amino acid sequences of the N-terminus of IgG were determined using a Procise 494 HT Protein Sequencing System (Applied Biosystems, Waltham, MA, USA) as previously published [12].

### 2.2. Biological Activity

To measure cAMP accumulation induced by recombinant AM-Fc-fusion proteins in vitro, we used HEK-293 cells stably expressing the AM type I receptor (AM_1_ receptor), which were prepared as previously described [15]. HEK-293 cells were cultured in Dulbecco’s modified Eagle’s medium supplemented with 10% fetal bovine serum, 100 U/mL penicillin G, 100 μg/mL streptomycin, 0.25 μg/mL amphotericin B, 100 μg/mL hygromycin B, and 250 μg/mL geneticin in a 24-well plate coated with human fibronectin (Thermo Fisher Scientific, Inc., Waltham, MA, USA) in a humidified 5% CO_2_ incubator maintained at 37 °C. After culturing for 3 days, cells at 90% confluency were subjected to experiments in which intracellular cAMP accumulation was stimulated using Fc-AMs. The culture media were replaced with Hanks’ Balanced Salt Solution containing 0.2% bovine serum albumin, 0.035% NaHCO_3_, and 0.5 mM isobutylmethylxanthine, and then cells were incubated with Fc-AMs for 15 min at 37 °C. The reactions were terminated by the addition of 0.1 M HCl. Finally, cAMP levels in supernatants were measured using an enzyme immunoassay kit (Cayman Chemical, Ann Arbor, MI, USA) followed by the protocol of Item No. 581002.

### 2.3. Pharmacokinetics

All male 7-week-old Wistar rats were purchased from Charles River Laboratories Japan (Kanagawa, Japan) and housed under a 12 h light/12 h dark cycle and specific pathogen-free conditions with a normal diet. We used 8 rats in this study. The study was performed in accordance with the Animal Welfare Act and with approval from the University of Miyazaki Institutional Animal Care and Use Committee (2019-533-2). To determine plasma IgG1-AM (6-52) and IgG4-AM (6-52) concentrations, 30 nmol/kg Fc-AMs were administered subcutaneously to rats (*n* = 2/group). Peripheral blood samples were drawn via the tail vein and placed into Kantan-tubes (Eiken Chemical Corp., Tochigi, Japan), containing heparin sodium, and then centrifuged at 2000× *g* for 3 min at room temperature. The plasma samples were then placed into tubes containing 21 μg aprotinin and 0.3 mg Na2-EDTA. Blood was collected before injection (day 0), and then 1, 2, 4, 6, 8, 10, 12, and 14 days after administration. Tissues were collected 7 and 14 days after administration, carefully resected after decapitation, and stored at −80 °C until use. The PK parameters (Vz/F, CL/F, T1/2, and Ke) were calculated with WinNonlin at the BoZo Research Center Inc. (Tsukuba, Japan). For the control measurement using AM, 50 nmol/kg native AM was administered subcutaneously (*n* = 3) and blood was collected before injection (0 min), and then 15, 30, 60 and 120 min after administration.

### 2.4. Sample Preparation for Enzyme-Linked Immuno Sorbent Assay (ELISA)

Tissue samples obtained after the administration of Fc-AMs were homogenized in PBS containing Protease Inhibitor Cocktail (Nacalai Tesque, Tokyo, Japan) using a Polytron mixer and immediately centrifuged at 20,000× *g* for 20 min at 4 °C. After centrifugation, the sample supernatant was used for ELISA. Two types of AM measurement systems were used for the measurement. As mature AM (mAM) [16] uses an antibody that recognizes the ring and C-terminus (AM (46-52) NH2), it is possible to measure active AM. However, total AM (tAM) [17] uses an antibody that recognizes the AM (25-36) and ring of AM. Therefore, assay for tAM can measure not only active AM but also inactive AM. Inactive AM is AM lacking the C-terminal region of AM or Fc-AM. Namely, the difference between tAM and mAM indicates the amount of inactive AM, in which active AM is cleaved by proteases. AM concentrations in tissues were corrected for total protein content measured using a Pierce BCA Protein Assay Kit (Thermo Fisher Scientific, Waltham, MA, USA).

### 2.5. Effect on Blood Pressure

Male 8-week-old spontaneously hypertensive rats (SHRs) were purchased from Charles River Laboratories (Kanagawa, Japan) and housed under a 12 h light/12 h dark cycle and specific pathogen-free conditions. IgG4 AM (6-52) (50 nmol/kg) or saline (control) was injected subcutaneously on the same day as the start of the administration of a high-salt diet (8% NaCl) (*n* = 5). The high-salt diet was given to rats until the final day of the experiment. Blood pressures of rats were measured using the tail-cuff method (model BP-98A; Softron, Tokyo, Japan), at the indicated time points (days 0, 3, 6, 9, and 12) according to the manufacturer’s instructions. The study was performed in accordance with the Animal Welfare Act and with the approval of the University of Miyazaki Institutional Animal Care and Use Committee (2019-527-2).

### 2.6. Statistical Analysis

**Figure 1:**
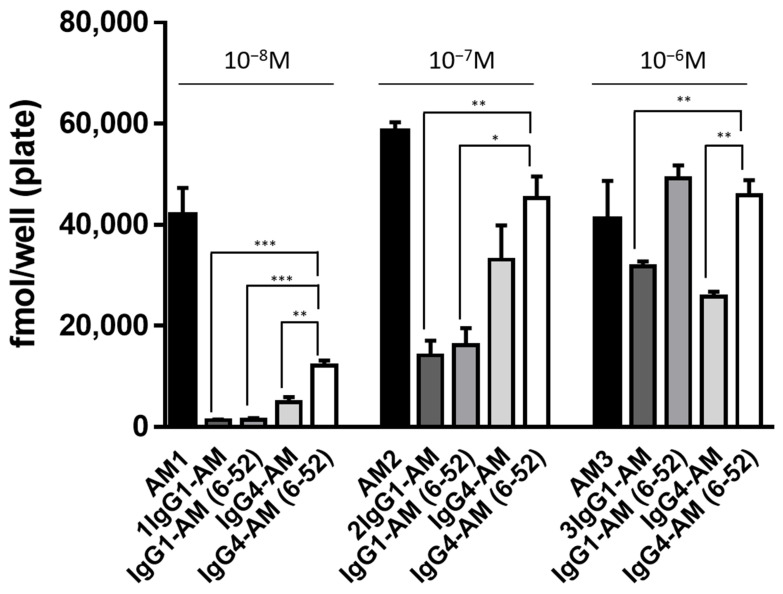
Intracellular cAMP accumulation induced by Fc-fusion proteins. HEK-293 cells stably expressing the AM_1_ receptor were incubated with AM or Fc-fusion proteins for 15 min, and the intracellular cAMP levels were measured with an enzyme immunoassay. The data were compared at concentrations of 10^−8^ M, 10^−7^ M, and 10^−6^ M, respectively. The experiments were repeated twice independently, and results are shown as means ± SD. * *p* < 0.05, ** *p* < 0.01, and *** *p* < 0.001 vs. IgG4-AM (6-52).

All data are presented as the mean ± SD. Statistical analysis was performed using GraphPad Prism 6 (GraphPad Software, Inc., La Jolla, CA, USA). Multiple comparisons were evaluated by one-way (Figure 1) and two-way (Figure 4a,b) ANOVA followed by Tukey’s multiple comparison test. Figure 4c,d were analyzed by the Mann–Whitney *U*-test. Values of *p* < 0.05 were considered statistically significant.

## 3. Results

### 3.1. Properties of Fc-AM

Each recombinant Fc-AM was purified from 1 L of supernatant as described in the Methods. The amount of tAM recovered per liter was 10.8 mg for IgG1-AM, 116.4 mg for IgG1-AM (6-52), 24.4 mg for IgG4-AM, and 12.0 mg for IgG4-AM (6-52), respectively, and 10.2, 108.1, 28.1, and 13.2 mg mAM, respectively, was recovered per liter. As the concentrations of tAM and mAM were similar, it was considered that all Fc-AMs were amidated. The molecular weights of recombinant proteins were estimated at approximately 66,500 Da by SDS-PAGE, corresponding to the dimeric forms of Fc-AM. These sequences were identical to the known amino acid sequences of human IgG1 and IgG4 as previously reported [12].

### 3.2. Intracellular cAMP Accumulation Induced by Fc-Fusion Proteins

We tested the biological activities of IgG1-AM, IgG1-AM (6-52), IgG4-AM, and IgG4-AM (6-52) to stimulate the intracellular accumulation of cAMP in HEK-293 cells stably expressing the AM_1_ receptor. As shown in Figure 1, the relative activities of IgG1-AM, IgG1-AM (6-52), IgG4-AM, and IgG4-AM (6-52) at 10^−8^ M were 3, 3, 12 and 29%, respectively, taking the maximum activity of native AM as 100%. Similarly, at 10^−7^ M, the relative activities were 25, 28, 57 and 77%, respectively (Figure 1). Derivatives bound to IgG4 tended to have higher cAMP-producing ability than those bound to IgG1. At 10^−6^ M, the relative activities were 77, 120, 63 and 111%, respectively (Figure 1). IgG1 and IgG4 tended to have higher cAMP production capacity when AM (6-52) was bound compared with AM. In addition, IgG4-AM (6-52) had the highest receptor binding ability among the four Fc-AMs.

### 3.3. Plasma Concentrations after a Single Subcutaneous Administration of IgG1-AM (6-52) or IgG4-AM (6-52) to Rats

**Figure 2:**
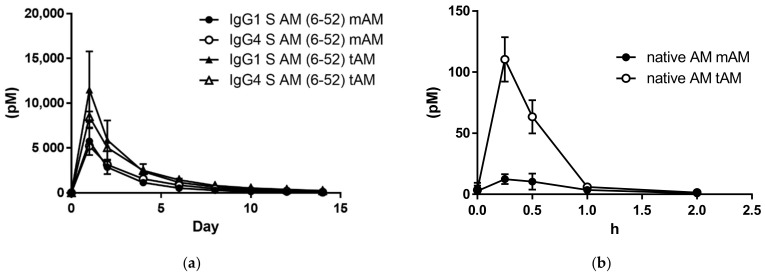
(**a**) Plasma disappearance of IgG1-AM (6-52) and IgG4-AM (6-52) over time. Concentrations of mature AM and total AM in plasma. Anesthetized rats were injected subcutaneously with IgG1-AM (6-52) (30 nmol/kg) and IgG4-AM (6-52) (30 nmol/kg), and blood samples were taken at the indicated time points. (**b**) Plasma disappearance of native AM over time. Native AM was injected subcutaneously into anesthetized rats at a dose of 50 nmol/kg, and blood samples were collected at the indicated time points. Concentrations of mature AM and total AM in plasma.

We compared IgG1-AM (6-52) and IgG4-AM (6-52) concentrations in the peripheral blood of rats after subcutaneous injection. After the administration of IgG1-AM (6-52), mAM concentrations in the blood after 0, 1, 2, 4, 6, 8, 10, 12, and 14 days were 31.2, 5757.4, 2865.0, 1145.6, 530.1, 281.1, 141.0, 81.5, and 34.0 pM, respectively (Figure 2a). Similarly, mAM concentrations in blood after the administration of IgG4-AM (6-52) were 0.1, 5220.1, 3135.1, 1561.8, 857.3, 452.0, 260.0, 185.5, and 81.3 pM, respectively (Figure 2a). Total AM concentrations at the same time points were 35.9, 11,499.8, 5892.1, 2376.1, 1162.8, 663.7, 391.3, 245.1, and 147.8 pM, respectively, after the administration of IgG1-AM (6-52) (Figure 2a). tAM concentrations in blood after the administration of IgG4-AM (6-52) were 0.5, 8486.3, 5040.4, 2484.5, 1433.5, 813.2, 517.0, 380.9, and 235.6 pM, respectively (Figure 2a). The changes in blood levels were similar for both Fc-AMs. The PK parameters for tAM and mAM are summarized in Table 1. The T1/2 of IgG1 S-AM (6-52) and IgG4 S-AM (6-52) was 2.11 days and 2.37 days, respectively, when mAM was monitored. When tAM was monitored, the T1/2 of IgG1 S-AM (6-52) and IgG4 S-AM (6-52) was 2.885 days and 3.23 days, respectively, which are slightly longer compared with those of mAM. Other parameters were similar for IgG1 S-AM (6-52) and IgG4 S-AM (6-52).

We further clarified the blood mAM and tAM concentrations after subcutaneous administration of AM (Figure 2b). The T1/2 of AM was 32.3 min when mAM was monitored. When tAM was monitored, the T1/2 of AM was 27.5 min. The Cmax concentration of tAM is higher than mAM.

### 3.4. Concentrations of IgG1-AM (6-52) and IgG4-AM (6-52) in Rat Tissues

**Figure 3:**
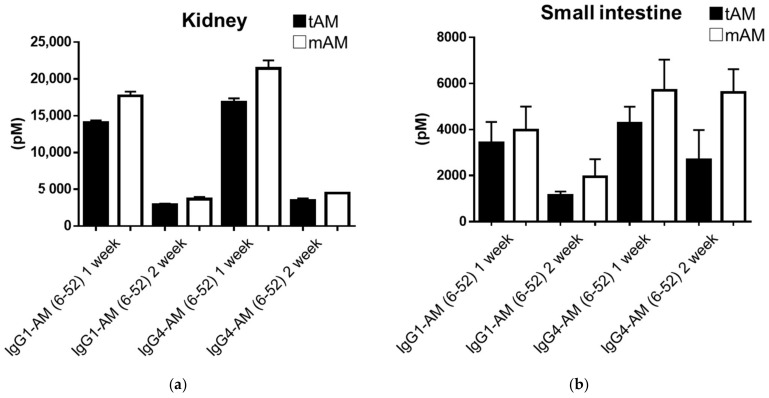
Concentrations of Fc-fusion protein in kidneys (**a**) and small intestines (**b**), 1 and 2 weeks after subcutaneous administration.

We compared IgG1-AM (6-52) and IgG4-AM (6-52) concentrations in the tissues of rats after subcutaneous injection. Table 2 and Table 3 show the concentrations of tAM and mAM in tissues. Fc-AM had the highest transferability to the kidney, followed by the small intestine (Figure 3). In addition, there was no difference in tAM and mAM levels between groups.

### 3.5. Hypotensive Effect of IgG4-AM (6-52) on SHRs

**Figure 4:**
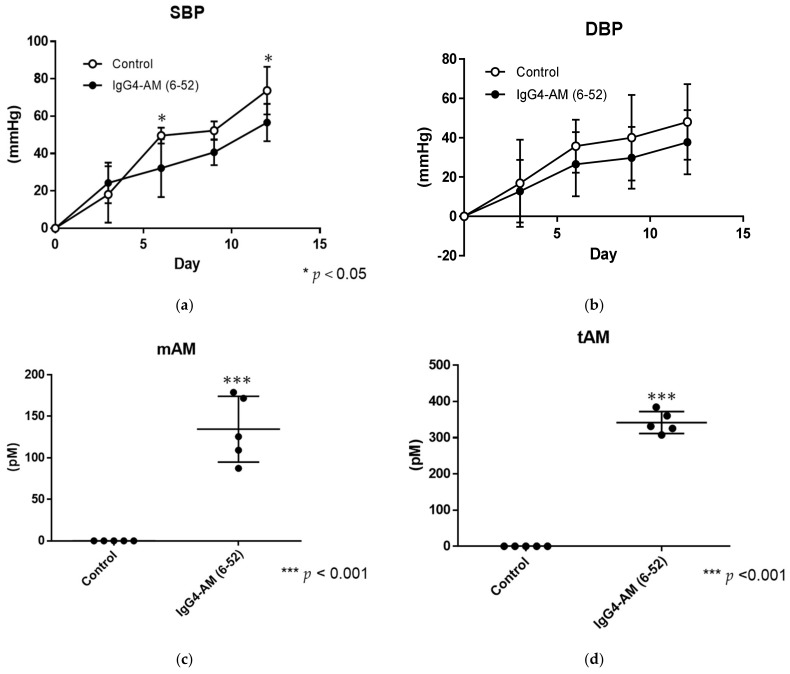
Blood pressure and plasma AM levels after treatment with IgG4-AM (6-52) (50 nmol/kg, *n* = 5). Data of SBP (**a**) and DBP (**b**) represent blood pressures at each timepoint following the administration, subtracted from blood pressures prior to administration: control group (open circles) and IgG4 AM (6-52) groups (closed circles). Levels of mature AM (**c**) and total AM (**d**) in the plasma after experiments. Results are shown as means ± SD. * *p* < 0.05, *** *p* < 0.001 vs. control.

Figure 4 shows the systolic blood pressures (SBPs) (Figure 4a) and diastolic blood pressures (DBPs) (Figure 4b) after the administration of IgG4-AM (6-52) to SHRs. Data represent blood pressures on days 1–12 following fusion protein administration, subtracted from blood pressures prior to administration. SBPs at 3, 6, 9, 12 days after treatment with IgG4-AM (6-52) were 24.3 ± 4.8, 32.2 ± 6.9, 40.7 ± 3.1, and 56.6 ± 4.5 mmHg, respectively (Figure 4a). SBPs of the control group were 18.1 ± 6.7, 49.6 ± 1.9, 52.3 ± 2.2, and 73.7 ± 5.7 mmHg, respectively (Figure 4a). The SBPs of SHRs treated with IgG4-AM (6-52) were significantly lower than those of controls on days 6 and 12.

DBPs at the same timepoints after treatment with IgG4-AM (6-52) were 12.8 ± 7.1, 26.5 ± 7.3, 29.8 ± 7.0, and 37.7 ± 7.3 mmHg, respectively (Figure 4b). DBPs in the control group were 16.9 ± 9.9, 35.7 ± 6.1, 40.0 ± 9.7, and 48.0 ± 8.6 mmHg, respectively (Figure 4b). There were no significant differences in DBP measurements between IgG4-AM (6-52) and control groups. We obtained plasma samples on day 12 after treatment and examined the mAM and tAM blood concentrations of SHRs in each group after the experiment. The mAM concentrations in the control and IgG4-AM (6-52) treated groups were 0.03 ± 0.03 and 134.6 ± 17.8 pM, respectively (*n* = 5) (Figure 4c). The tAM concentrations in the control and treatment groups were 0.05 ± 0.02 and 341.9 ± 13.6 pM, respectively (*n* = 5) (Figure 4d).

## 4. Discussion

We developed Fc-AMs, which are long-acting AM derivatives. We previously produced Fc-fusion AM proteins by genetic recombination in *E. coli* [12]. However, AM produced in *E. coli* requires refolding and amidation, whereas Fc-AMs produced in mammalian cells do not require refolding or amidation. Indeed, Fc-AMs could be measured by mAM assay, which recognizes the amidated form, indicating that Fc-AM produced in mammalian cells were amidated.

Fc-AMs, generated in a mammalian cell production system can therefore easily be produced in large amounts with few purification steps. This system reduces their manufacturing costs, so it may be possible to provide them to many patients. The recombinant fusion of IgG Fc fragments and peptides, enzymes or cytokines has been recognized as a new therapeutic strategy [18]. Indeed, most IgG Fc fusions are produced in mammalian cells, such as CHO and HEK cells. Dulaglutide—a GLP-1 receptor agonist (IgG4 Fc-fusion GLP-1)—is used for the treatment of type 2 diabetes mellites [19]. Dulaglutide was also produced using HEK-293 cells.

The biological effects of AM are mediated by the calcitonin receptor-like receptor (CLR), which can function as an AM type 1 or 2 receptor when co-expressed with receptor activity-modifying protein-2 (RAMP2) and -3 (RAMP3) [20]. The Fc-fusion proteins stimulated intracellular cAMP production in cultured cells stably expressing the AM type I receptor (CLR/RAMP2) in vitro (Figure 1). These Fc-fusion proteins tended to have higher cAMP production capacity when AM (6-52) was bound than when full-length AM was bound. These results were similar to those of Fc-AMs prepared using *E. coli* in a previous study [12]. Interestingly, IgG4 fusion proteins had higher cAMP-producing ability than IgG1 fusion proteins (Figure 1). These differences may be explained by structural changes, stability, and various other factors. However, it was difficult to clarify these reasons in the present study and additional experimentation will be necessary.

From the results of cAMP production (Figure 1), a pharmacokinetic study was performed using N-terminal deficient AM derivatives, which have strong receptor binding ability. A comparison analysis showed the activity of Fc-AM was approximately 10% that of AM, although the exact EC50 was not determined in the present study. According to previous Phase I and Phase IIa clinical trials [21,22], the blood concentration of AM was about 10 pM when AM was administered continuously and efficacy was observed. Therefore, sufficient concentrations of IgG1-AM (6-52) and IgG4-AM (6-52) might be present in blood 14 days after a single subcutaneous administration. The pharmacokinetics of IgG1-AM (6-52) and IgG4-AM (6-52) were similar and a sufficient amount of active mAM was observed in the blood until 14 days post-administration (Figure 2a).

As expected, Fc-AMs were less active in the cAMP assay compared with AM. We produced various analogs of PEG-AM whose N-terminus was attached to various sizes of PEG [23,24]. We found that the activity decreased as the molecular weight of PEG-AM increased. Compared with PEG-AM, Fc-AM activity was within the expected range. Although the exact mechanism of the decreased activity of Fc-AM in the cAMP assay is unclear, Fc bound to the N-terminus of AM may interfere with the binding of Fc-AM to the AM receptor.

When comparing the activities of AM and Fc-AM, the maximal activities at high concentrations were equivalent. Therefore, we think that a higher concentration of Fc-AM might exert the same effect as AM. As AM has a high affinity for the AM receptor, it causes rapid hypertensive effects when it is administered as a bolus. However, Fc-AM is expected to have no rapid antihypertensive effects and therefore be a safe drug, as in the case of PEG-AM [25].

Sufficient concentrations of IgG1-AM (6-52) and IgG4-AM (6-52) were observed in blood 14 days after a single subcutaneous administration. The pharmacokinetics of IgG1-AM (6-52) and IgG4-AM (6-52) were similar (Figure 2a). We have calculated the PK parameters and summarized them as Table 1. The half-life of IgG1 S-AM (6-52) and IgG4 S-AM (6-52) was 2.11 days and 2.37 days, respectively, when mAM was monitored. These values seem to be much longer compared to the half-life of AM in humans and rats, as reported [6,21,25]. A sufficient amount of active mAM was observed in the blood until 14 days post-administration. This might be explained by the fact that IgG Fc fusions with bioactive peptides have a longer plasma half-life associated with their large size. Alternatively, intracellular neonatal Fc receptor-mediated recycling may suppress intracellular degradation [26]. In this way, Fc-AM is thought to have properties not found in other derivatives, but further research is needed to elucidate this.

In the pharmacokinetic study, the plasma concentrations of Fc-AMs were high, even after 2 weeks; therefore, we investigated their transferability to tissues. As shown in Table 1, Fc-AMs were present in many tissues, and high concentrations of Fc-AM were detected in the kidneys and small intestines. Concentrations of mAM in tissues, which is the active form of AM, remained comparable to those of tAM. In a PET imaging study, a NOTA (1,4,7-triazacyclononane-1,4,7-triacetic acid)-conjugated AM analog migrated to the lungs and kidneys [27]. Although the molecular weight was different from that of Fc-AM, it had a similar migratory ability to the kidneys and lungs. Therefore, it is reasonable that IgG1-AM and IgG4-AM are abundant in the lungs and kidneys. High concentrations of IgG1-AM and IgG4-AM were unexpected in the small and large intestines. These data suggest that Fc-AM is more effective in diseases of the kidney and intestine. Of note, a high concentration of IgG1-AM (6-52) was observed in the large intestine for mAM but not tAM. In addition, a high concentration of mAM but not tAM was found in the small intestine after the administration of IgG4-AM (6-52). We cannot explain this finding, but the antigenicity of Fc-AM might change depending on its conformation or molecular form. The AM_1_ receptor is expressed by a variety of tissues, including the kidneys and small intestines [28]. However, detected Fc-AM might not always be bound to the receptor and Fc-AM may be more effective in diseases of the kidney and small intestine. It was reported that AM binds to complement factor H, which is abundant in plasma [29]. Although Fc-AM may also bind to complement factor H in tissues, further study to determine which protein binds to Fc-AM in tissues is necessary.

We further investigated the pharmacokinetics of AM after the subcutaneous injection of 50 nmol/kg of AM. The peak mAM concentration was 12.5 pM for AM. This value is much lower compared to those of IgG1 S-AM (6-52) and IgG4 S-AM (6-52). In addition, the plasma half-life of IgG1 S-AM (6-52) and IgG4 S-AM (6-52) was much longer and the Tmax was much later than that of AM (Figure 2a,b). Thus, Fc-AM will likely prove useful as a drug for treating several disorders, for which AM is reported to be effective.

Interestingly, in Figure 2b, the concentration of tAM was higher than that of mAM for AM. The assay for mAM can only measure the active AM, while the assay for tAM can measure both the active and inactive AM, with the latter lacking the C-terminal region. Therefore, the higher concentration of tAM compared with that of mAM suggests that AM is cleaved extensively by endogenous proteases. In contrast, tAM and mAM were almost equivalent in concentration following the subcutaneous injection of IgG1 S-AM (6-52) and IgG4 S-AM (6-52), indicating that they are more resistant to endogenous proteases.

In SHR, IgG4-AM (6-52) suppressed increased blood pressure, especially the SBP. However, there were no significant differences in DBP measurements between the IgG4-AM (6-52) and control groups. Thus, IgG4-AM (6-52) can partially inhibit the increase in SBP increase in SHRs. These effects may be related to the functions of native AM, including vasodilation and suppressive effects on aldosterone and vasopressin secretion [30]. In addition, even after 12 days, sufficient amounts of Fc-AM remained, indicating that the effects lasted for 2 weeks, which was associated with its long half-life in blood. Therefore, Fc-AM may be useful for chronic diseases that require long-term treatment. Moreover, Fc-AM can be produced in large amounts in a few steps, and IgG and AM are endogenous molecules in the human body. As it is safe, inexpensive, and requires a small number of administrations, it might be available to many patients. AM was effective in a variety of disease model animals, including inflammatory bowel disease [2], ischemic heart disease [3], sepsis, and stroke [4]. Therefore, it might be applied to diseases for which treatment has been reported in AM.

Although Fc-AM is a promising therapeutic drug for various diseases including cardiovascular and inflammatory diseases, there were some limitations in this study. AM can activate three distinct receptors (CGRP, AM_1_, and AM_2_ receptors) [31]. However, in this study, we examined the effect of Fc-AM on the AM_1_ receptor but not the CGRP or AM_2_ receptors. We think that Fc-AM may activate the CGRP and AM_2_ receptors. The effect of Fc-AM on CGRP or AM_2_ receptors should be examined in the future. Another limitation was that the sample size was small in the PK experiments, although the data are clear. We could not increase the sample size, as the amount of AM-Fc-fusion protein was limited. Fc-fusion proteins are easily produced in large amounts by industrial methods; however, the preparation of Fc-fusion proteins is difficult and limited in the laboratory. Therefore, the PK experiments of Fc-AM derivatives in this study were preliminary.

## 5. Conclusions

Fc-AM produced from mammalian cells was easily produced and might be a candidate novel therapeutic agent.

## Figures and Tables

**Table 1:** The PK parameters (Vz/F, CL/F, T1/2, and Ke) and concentrations of mAM and tAM.

mAM			tAM		
	IgG1 S AM (6-52)	IgG4 S AM (6-52)		IgG1 S AM (6-52)	IgG4 S AM (6-52)
t1/2 (days)	2.110	2.370	t1/2 (days)	2.885	3.235
Elimination rate constant (K) (1/days)	0.332	0.296	Elimination rate constant (K) (1/days)	0.240	0.216
Vz/F (L/kg)	6.640	6.065	Vz/F (L/kg)	4.360	5.370
CL/F (L/day/kg)	2.135	1.775	CL/F (L/day/kg)	1.047	1.165
Day	Raw data (pM)		Day	Raw data (pM)	
0	31.2	0.1	0	35.9	0.5
1	5757.4	5220.1	1	11,499.8	8486.3
2	2865.0	3135.1	2	5892.1	5040.4
4	1145.6	1561.8	4	2376.1	2484.5
6	530.1	857.3	6	1132.8	1433.5
8	281.1	452.0	8	663.7	813.2
10	141.0	260.0	10	391.3	517.0
12	81.5	185.5	12	245.1	380.9
14	34.0	81.3	14	147.8	235.6

**Table 2:** The tissue levels of tAM were determined by enzyme immunoassay.

tAM	IgG1-AM (6-52)		IgG4-AM (6-52)	
(pmol/g protein)	Day 7	Day 14	Day 7	Day 14
Brain	2.49	1.23	2.10	0.90
Lung	7.66	3.73	10.37	2.13
Heart	4.22	1.57	4.21	1.37
Kidney	53.53	10.93	42.05	8.63
Adrenal gland	2.68	1.52	2.83	1.10
Liver	0.46	0.12	0.31	0.13
Pancreas	1.79	0.48	0.59	0.21
Spleen	1.42	0.40	1.37	0.48
Large intestine	4.74	1.27	4.20	0.92
Small intestine	12.95	4.30	10.69	6.73
Stomach	3.38	0.59	2.24	0.98

**Table 3:** The tissue levels of mAM were determined by enzyme immunoassay.

mAM	IgG1-AM (6-52)		IgG4-AM (6-52)	
(pmol/g protein)	Day 7	Day 14	Day 7	Day 14
Brain	2.07	1.14	1.67	0.75
Lung	5.82	3.46	8.30	1.67
Heart	3.55	1.58	3.29	1.10
Kidney	76.05	15.86	57.86	12.06
Adrenal gland	3.63	2.29	2.54	1.29
Liver	0.30	0.11	0.22	0.11
Pancreas	1.55	0.51	0.68	0.34
Spleen	0.98	0.37	1.00	0.36
Large intestine	26.79	1.48	6.00	1.48
Small intestine	17.09	8.40	15.41	15.15
Stomach	4.75	0.64	2.61	1.17

## Data Availability

Not applicable.
